# Response to Comment on: Revision Total Knee Arthroplasty Using Robotic Arm Technology

**DOI:** 10.1016/j.artd.2022.101091

**Published:** 2023-01-31

**Authors:** Micah MacAskill, Baylor Blickenstaff, Alexander Caughran, Matthew Bullock

**Affiliations:** Marshall University, Joan C Edwards, School of Medicine, Huntington, WV, USA

The authors of “Revision Total Knee Arthroplasty Using Robotic Arm Technology” [1] would like to acknowledge the editors request for comment on a publishing concern regarding our article. We would like to congratulate Steelman et al [2] for being the first to publish on the topic in August 2021. Well before our article’s submission in September 2021, we performed an exhaustive literature search and were unable to locate the article by Steelman et al [2]. One must keep in mind the publishing delay due to the editing process once an article is submitted. Second, there is a known delay in PubMed indexing after an article is published [3]. At the time of our submission, “to our knowledge, there are no reports of robotic technology being used for the revision of a total knee arthroplasty” [1].

We have reviewed the article by Steelman et al [2] and openly appreciate their contributions to the field of robotic revision arthroplasty. Our case series is the result of extensive problem solving and critical thinking required to summarize our findings to maximize registration and modeling accuracy. Considering both articles, there are now currently 3 cases of knee revision using robotic-arm technology within the published literature with more to come. We acknowledge that “Utilization of Robotic Arm Assistance for Revision of Primary Total Knee Arthroplasty: A Case Report” was published ahead of our case series [2]. Thank you.

## Conflicts of interest

Matthew Bullock is a paid presenter for Smith & Nephew; is an unpaid consultant for Osso VR; has stock in Stryker; received educational support from Stryker, Smith & Nephew, Depuy, and Zimmer/Biomet; is a part of Editorial Board Arthroplasty Today; is a board member of AAKHS Patient Education Committee and West Virginia Orthopaedic Society Education Committee. The other authors declare no potential conflicts of interest.

For full disclosure statements refer to https://doi.org/10.1016/j.artd.2022.101091.

## Appendix A Supplementary Data

Conflict of Interest Statement for all the Authors
